# Medicine a State Institution in Spain

**Published:** 1855-01

**Authors:** 


### *Medicine a State Institution in Spain.*

The Gazette publishes a Royal Decree to the following effect:

"Art. 1. Every town and locality in the kingdom are in future to be provided with physicians, surgeons and apothecaries, whose duty it will be to dispense medical aid to the indigent classes, and any other persons who may require their attendance.

" Art. 2. The existence of these physicians shall not prevent the free exeicise of the medical profession in the same localities.

" Art. 3. The authorities will maintain in the free exercise of their profession, the persons who have been legally accredited, in virtue of the present decree, and other ordinances in force.

" Art. 4. The physicians, independently of their attendance on the sick, will have to take charge of foundlings, to decide whether substitutes are fit for the militaiy service, and to visit sick soldiers passing through their districts. They are not to absent themselves from the town in which they practice during more than twenty-four hours without the permission of the Alcalde, and for a longer period without providing a substitute. Their salary is to be proportioned to the population of the district, the wealth of its inhabitants, and other local ci?cumstances. They will be entitled to a pension after practising thirty years in the same district.".—*London Times.*

